# Genetic and Epigenetic Alterations of *CDH1* Regulatory Regions in Hereditary and Sporadic Gastric Cancer

**DOI:** 10.3390/ph14050457

**Published:** 2021-05-12

**Authors:** Gianluca Tedaldi, Chiara Molinari, Celina São José, Rita Barbosa-Matos, Ana André, Rita Danesi, Valentina Arcangeli, Mila Ravegnani, Luca Saragoni, Paolo Morgagni, Francesca Rebuzzi, Matteo Canale, Sara Pignatta, Elisa Ferracci, Giovanni Martinelli, Guglielmina Nadia Ranzani, Carla Oliveira, Daniele Calistri, Paola Ulivi

**Affiliations:** 1 Biosciences Laboratory, IRCCS Istituto Romagnolo per lo Studio dei Tumori (IRST) “Dino Amadori”, 47014 Meldola, Italy; chiara.molinari@irst.emr.it (C.M.); francesca.rebuzzi@irst.emr.it (F.R.); matteo.canale@irst.emr.it (M.C.); sara.pignatta@irst.emr.it (S.P.); daniele.calistri@irst.emr.it (D.C.); paola.ulivi@irst.emr.it (P.U.); 2 i3S, Institute for Research and Innovation in Health, University of Porto, 4200-135 Porto, Portugal; cjose@ipatimup.pt (C.S.J.); amatos@ipatimup.pt (R.B.-M.); aandre@ipatimup.pt (A.A.); 3 IPATIMUP, Institute of Molecular Pathology and Immunology, University of Porto, 4200-135 Porto, Portugal; 4 Doctoral Programme in Biomedicine, Faculty of Medicine, University of Porto, 4200-319 Porto, Portugal; 5 Doctoral Programme on Cellular and Molecular Biotechnology Applied to Health Sciences (Biotech Health), ICBAS-Institute of Biomedical Sciences Abel Salazar, University of Porto, 4050-313 Porto, Portugal; 6 Romagna Cancer Registry, IRCCS Istituto Romagnolo per lo Studio dei Tumori (IRST) “Dino Amadori”, 47014 Meldola, Italy; rita.danesi@irst.emr.it (R.D.); valentina.arcangeli@irst.emr.it (V.A.); mila.ravegnani@irst.emr.it (M.R.); 7 Pathology Unit, Morgagni-Pierantoni Hospital, 47121 Forlì, Italy; luca.saragoni@auslromagna.it; 8 Department of General Surgery, Morgagni-Pierantoni Hospital, 47121 Forlì, Italy; paolo.morgagni@auslromagna.it; 9 Laboratory of Stem Cells and Cancer Genomics, University of Trento, 38123 Trento, Italy; elisa.ferracci@unitn.it; 10 Department of Medical Oncology, IRCCS Istituto Romagnolo per lo Studio dei Tumori (IRST) “Dino Amadori”, 47014 Meldola, Italy; giovanni.martinelli@irst.emr.it; 11 Department of Biology and Biotechnology, University of Pavia, 27100 Pavia, Italy; guglielmina.ranzani@unipv.it; 12 Faculty of Medicine of the University of Porto (FMUP), 4200-319 Porto, Portugal

**Keywords:** gastric cancer, *CDH1* gene, DNA methylation, regulatory regions, genetic predisposition, Next-Generation Sequencing

## Abstract

E-cadherin is a key player in gastric cancer (GC) and germline alterations of *CDH1*, its encoding gene, are responsible for Hereditary Diffuse Gastric Cancer (HDGC) syndrome. This study aimed at elucidating the role of genetic variants and DNA methylation of *CDH1* promoter and enhancers in the regulation of gene expression. For this purpose, we analyzed genetic variants of the *CDH1* gene through Next-Generation Sequencing (NGS) in a series of GC cell lines (NCI-N87, KATO-III, SNU-1, SNU-5, GK2, AKG, KKP) and the corresponding *CDH1* expression levels. By bisulfite genomic sequencing, we analyzed the methylation status of *CDH1* regulatory regions in 8 GC cell lines, in a series of 13 sporadic GC tissues and in a group of 20 HDGC *CDH1*-negative patients and 6 healthy controls. The NGS analysis on *CDH1* coding and regulatory regions detected genetic alterations in 3 out of 5 GC cell lines lacking functional E-cadherin. *CDH1* regulatory regions showed different methylation patterns in patients and controls, GC cell lines and GC tissues, expressing different E-cadherin levels. Our results showed that alterations in terms of genetic variants and DNA methylation patterns of both promoter and enhancers are associated with *CDH1* expression levels and have a role in its regulation.

## 1. Introduction

In 2020, gastric cancer (GC) ranked fifth for incidence and fourth for mortality, worldwide [1]. Although GC is commonly sporadic, about 10–20% of cases show familial clustering and 1–3% of cases can be considered hereditary [2]. Sporadic GC occurs at an older age, is mainly associated with intestinal histotype (IGC) [3] and is related to environmental risk factors, such as microbial infection and diet [4]. Hereditary GC is characterized by early-onset, is mainly of the diffuse histotype (DGC) and is associated with alterations in different predisposition genes [5]. Nevertheless, with the exception of the inherited familial syndromes, the observed familial clustering of cancer is not well explained and may occur due to an inherited genetic susceptibility, together with shared lifestyle and environmental factors [6].

The main gene involved in hereditary GC is *CDH1*, encoding the E-cadherin protein [7], whose germline mutations are responsible for Hereditary Diffuse Gastric Cancer (HDGC) [8], a genetic syndrome linked to a lifetime GC risk of 42–70% in male carriers and 33–56% in female carriers, who also have a 39–55% risk of lobular breast cancer (LBC) [9,10]. The oncogenesis in HDGC syndrome follows the “two-hit model” [11], in which the presence of a germline mutation of the *CDH1* gene (first hit) is followed by the inactivation of the other allele (second hit) by mutations or deletions (loss of heterozygosity-LOH). However, it has been shown that *CDH1* promoter hypermethylation represents the main cause of inactivation of the wild-type allele in HDGC tumors [12,13,14].

The *CDH1* gene is a driver of carcinogenesis also in sporadic GC of both diffuse and intestinal histotypes [15,16] and *CDH1* epigenetic alterations, such as promoter hypermethylation and histone modifications, have also been detected in most sporadic GCs as a cause of E-cadherin impairment [15,17,18,19,20].

Given the importance of *CDH1* in the oncogenesis of hereditary and sporadic GC, different studies were carried out to understand the regulatory mechanisms of *CDH1* gene expression. In particular, the studies focused on the 65kb-region of *CDH1* intron 2, which is characterized by the presence of cis-regulatory sequences necessary for transcription and, therefore, for the regulation of gene expression [21,22,23].

Moreover, not only a large body of data supports the notion that DNA methylation is crucial in regulating gene expression, but recent findings also point to a link between this mechanism and cancer predisposition. Accordingly, studies aimed at linking genomic regions associated with cancer susceptibility and expression of genes involved in cancer development have already shown that promoters’ and enhancers’ sequence polymorphisms and methylation status can contribute to cancer risk [24,25].

The present study aimed to perform a molecular characterization of genetic and epigenetic alterations of *CDH1* promoter and enhancer sequences to deeply understand the mechanisms that regulate gene expression and to provide the rationale for investigating *CDH1* methylation in *CDH1*-negative patients with suspected genetic predisposition to GC.

## 2. Results

### 2.1. CDH1 Variant Analysis on GC Cell Lines

The *CDH1* variant analysis was performed by Next-Generation Sequencing (NGS) on seven GC cell lines (NCI-N87, KATO-III, SNU-5, SNU-1, AKG, GK2, KKP).

Results showed the presence of a *CDH1* pathogenic variant in three cell lines (KATO-III, SNU-5 and KKP), whereas in the other four cell lines no *CDH1* variants with relevant significance were detected (Supplementary Table S1).

### 2.2. Identification of CDH1 Regulatory Regions

**Figure 1:**
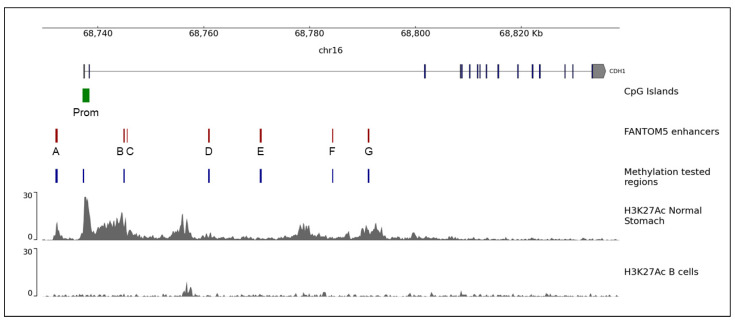
Scheme of the *CDH1* gene (chr16) with the CpG island represented by the green bar and enhancers by red bars. Blue bars represent the regions tested for methylation in the present work. The lower panels represent H3K27Ac, a chromatin mark associated with transcription activation [26], in normal stomach and B cells, respectively. The figure was generated with pyGenomeTracks [27].

The promoter and the enhancers of the *CDH1* gene were identified through the UCSC Genome Browser. The *CDH1* promoter is located in a CpG island of 1310 bp covering exons 1 and 2 (Figure 1). The FANTOM5 tool of the UCSC Genome Browser identified seven enhancers in the *CDH1* locus, one located 4724 bp upstream from the *CDH1* transcription start site (TSS), and six located within intron 2 (Figure 1).

### 2.3. CDH1 Methylation Analysis on DNA from GC Cell Lines, Peripheral Blood and Gastric Tissues

The methylation analysis of *CDH1* promoter and enhancers was performed on eight GC cell lines (NCI-N87, KATO-III, SNU-5, SNU-1, AKG, GK2, KKP and MKN-74), on germline DNA from peripheral blood of 6 healthy individuals and 20 HDGC *CDH1*-negative patients (15 with DGC and 5 with LBC) and on gastric normal and tumor tissues from 13 sporadic GC patients (10 with IGC, 1 with DGC, 1 with mucinous GC and 1 with mixed GC). We analyzed the methylation status of CpG sites located in the core of the *CDH1* promoter and in six enhancers of *CDH1* gene (Figure 1 and Supplementary Table S2).

**Figure 2:**
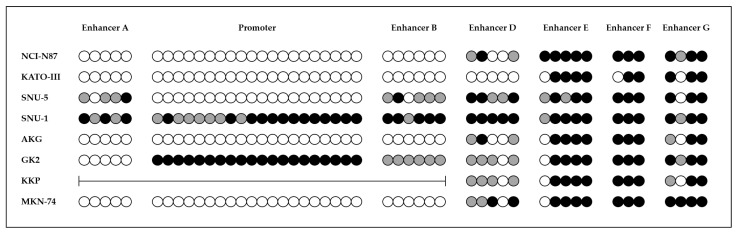
Methylation status of *CDH1* promoter and enhancers of eight GC cell lines expressing different levels of *CDH1*. 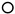 non-methylated CpG site; 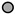 partially methylated CpG site; 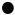 fully methylated CpG site; 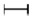 deletion.

The methylation analysis revealed different methylation patterns in the eight GC cell lines, with different CpG sites being methylated along promoter and enhancer sequences, in particular enhancers A, B and D (Figure 2).

**Figure 3:**
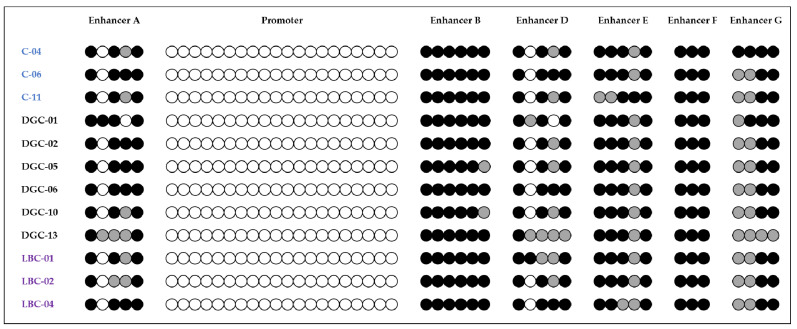
Methylation analysis of *CDH1* promoter and enhancers on a selection of DNA samples from peripheral blood of three healthy individuals (in blue) and nine HDGC *CDH1*-negative patients (six DGC patients in black and three LBC patients in violet). The complete case series is reported in Supplementary Figures S1 and S2. 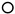 non-methylated CpG site; 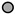 partially methylated CpG site; 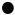 fully methylated CpG site.

The methylation analysis performed on the germline DNA derived from blood of 6 healthy controls and of 20 HDGC *CDH1*-negative patients (15 with DGC and 5 with LBC) revealed a strongly conserved methylation pattern along promoter and enhancer sequences, with absence of methylation in the promoter and almost full methylation in enhancer B in all patients and controls (Figure 3 and Supplementary Figures S1 and S2).

**Figure 4:**
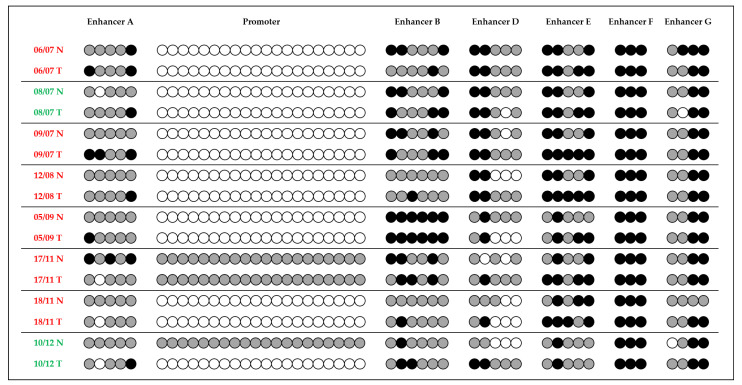
Methylation analysis of *CDH1* promoter and enhancers on DNA samples from gastric normal (N) and tumor (T) tissues from eight IGC patients (GC tissues with *CDH1* downregulation in red and GC tissues with *CDH1* upregulation in green). 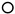 non-methylated CpG site; 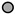 partially methylated CpG site; 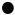 fully methylated CpG site.

The methylation analysis performed on DNA from gastric normal and tumor tissues of 13 patients with sporadic GC revealed differences in the methylation pattern of normal vs. tumor tissues, with an increased methylation in particular in enhancers A, B and E of tumor tissues (Figure 4 and Supplementary Figure S3).

### 2.4. CDH1 Expression Analysis in GC Cell Lines and Gastric Tissues

The *CDH1* expression analysis was performed by quantitative PCR (qPCR) on seven GC cell lines (NCI-N87, KATO-III, SNU-5, SNU-1, AKG, GK2, KKP) and on gastric normal and tumor tissues of eight sporadic IGC patients.

Results from GC cells revealed a reduced *CDH1* expression in four of them (SNU-1, SNU-5, GK2 and KKP) and a “normal” *CDH1* expression in the other three cell lines (NCI-N87, KATO-III and AKG) (Supplementary Table S3).

Results from gastric normal vs. tumor tissues revealed *CDH1* downregulation in six IGC tissues (06/07, 09/07, 12/08, 05/09, 17/11 and 18/11) and *CDH1* upregulation in two IGC tissues (08/07 and 10/12) (Table 1).

## 3. Discussion

GC is one of the most aggressive cancers, with high mortality rates worldwide. For this reason, the prevention and treatment of GC represent the main topics of interest in the research on this disease. One of the most important players in carcinogenesis of both hereditary and sporadic GC is the *CDH1* gene, encoding E-cadherin, a component of the adherent junctions between epithelial cells with a tumor suppressor activity [28].

In the present study, we evaluated the role of genetic and epigenetic alterations of the *CDH1* gene, in particular the methylation of promoter and enhancers, in the regulation of gene expression and in the predisposition to the development of GC.

Indeed, studies aimed at linking genomic regions associated with cancer susceptibility and expression of genes involved in cancer development have already shown that promoters’ and enhancers’ sequence polymorphisms and methylation status can contribute to cancer risk [24,25].

This evidence prompted us to test this model in the *CDH1* gene on GC cell lines and different tissues from patients with hereditary and sporadic GC.

Initially, we identified regulatory regions of the *CDH1* gene using bioinformatics tools for the identification of genomic regions with enhancer properties. This analysis revealed that, besides the promoter, there are seven regions in the *CDH1* gene with regulatory functions and six of them are located in intron 2, which has been previously identified as a cis-regulatory element with a pivotal role in controlling the gene expression [22].

Consequently, we explored the landscape of the *CDH1* gene in terms of DNA mutation and methylation on a series of eight GC cell lines, correlating these results to the gene expression. In particular, we identified *CDH1* pathogenic variants in three GC cell lines (KATO-III, SNU-5, KKP). Interestingly, SNU-5 and KKP cell lines also had a significant downregulation of *CDH1* expression. KKP cell lines showed a homozygous deletion of a large region including the promoter (with exons 1 and 2) and enhancers A and B, located upstream and downstream, respectively, while the methylation status of the other enhancers (D–G) did not show significant differences compared to GC cell lines expressing *CDH1*. The SNU-5 cell line showed a *CDH1* variant immediately after exon 5 affecting splicing and partial methylation of enhancers A, B and D (absent in the promoter). Of note, these two cell lines are derived from ascitic effusion of patients with a poorly differentiated GC. On the other hand, the third *CDH1*-mutated GC cell line, KATO-III, despite the presence of a variant in exon 7 with a deleterious effect on splicing [29], showed neither decreased *CDH1* expression nor increased promoter/enhancer methylation, suggesting that this variant does not alter the transcription, but rather the protein function. Indeed, KATO-III cell line is derived from a DGC with signet ring cells, a typical characteristic of GCs of HDGC patients.

The methylation analysis on the *CDH1* promoter and enhancers revealed increased methylation levels also in SNU-1 and GK2 cell lines, expressing low *CDH1* levels but lacking *CDH1* variants. Intriguingly, GK2 cells show full methylation of the *CDH1* promoter and partial methylation of enhancers B and D (but absent in enhancer A). On the other hand, SNU-1 cells show medium-high levels of methylation in promoter and enhancers A, B and D, compared to GC cell lines expressing *CDH1*. Of note, these two cell lines are both derived from poorly differentiated GCs.

The last three cell lines (NCI-N87, AKG and MKN-74) showed methylation neither in *CDH1* promoter nor in enhancers A and B. Indeed, these GC cell lines have a “normal” expression of *CDH1* gene, as demonstrated in the present work (for NCI-N87 and AKG) or previously reported (for MKN-74) [30,31]. Of note, these three GC cell lines are all derived from IGCs well or moderately differentiated.

Overall, the methylation results obtained in GC cell lines suggest that, besides the promoter, the methylation of CpG sites located in the *CDH1* enhancers correlates with the gene expression (Table 2).

These results prompted us to analyze the *CDH1* promoter/enhancer methylation in the germline DNA of HDGC *CDH1*-negative patients, in order to identify a signature predisposing GC development in these patients. Indeed, an increased methylation of *CDH1* promoter was previously identified in an HDGC *CDH1*-negative family [32].

To study the methylation of *CDH1* promoter and enhancers in the germline DNA, we analyzed the methylation on DNA extracted from peripheral blood on a series of 6 healthy individuals and 20 HDGC *CDH1*-negative patients (15 with DGC and 5 with LBC). Unexpectedly, this analysis showed a strongly conserved methylation pattern in *CDH1* promoter/enhancers both in the healthy controls and the HDGC patients. In particular, enhancer B showed a strong methylation pattern, together with enhancers A and D that were characterized by the presence of CpG sites with partial or full methylation.

E-cadherin, being a component of cell junctions, is not expressed in blood cells but the absence of methylation in the promoter suggests that another mechanism is responsible for *CDH1* downregulation. Our results highlight that, in blood cells, the methylation of *CDH1* enhancers could be responsible for the lack of expression of the gene. On the other hand, the methylation analysis of promoter/enhancers did not prove to be a valid method to identify individuals with a genetic predisposition to GC. Although DNA methylation is generally not heritable, alterations of the mechanisms underlying DNA methylation can, instead, be inherited, for example, in the form of germline mutations in genes involved in the DNA methylation/demethylation processes. However, considering our results, this event seems not to be a frequent cause of HDGC syndrome.

Finally, we tested the methylation levels of *CDH1* promoter/enhancers on a series of sporadic GCs. Results obtained from the analysis of eight IGCs expressing different levels of *CDH1* gene showed the presence of some differences in the methylation of *CDH1* promoter/enhancers between normal and tumor tissues. One of the two tumors (10/12) with upregulation of *CDH1* gene showed a decrease in the promoter methylation compared to the normal tissue, whereas the other (08/07) was characterized by a methylation status substantially comparable with the normal tissue, with some CpG sites of enhancers D and G less methylated in the tumor tissue.

Regarding the six tumors with downregulation of *CDH1* gene, they were characterized by the absence of promoter methylation in all normal and tumor tissues, with the exception of one case (17/11) that showed partial methylation of all the CpG sites analyzed in both normal and tumor tissues. On the other hand, the methylation status of *CDH1* enhancers showed slight differences between normal and tumor tissues, with an increased methylation especially in the CpG sites of enhancers A, B and E in almost all the cases. An additional series of five patients with sporadic GC was studied for *CDH1* promoter, enhancer A and B methylation (Supplementary Figure S3) showing a similar pattern.

This result suggests that *CDH1* enhancer methylation could have a role in gene expression regulation also in GC of the intestinal histotype, in which E-cadherin impairment is a less frequent event [15]. Moreover, in IGC, the *CDH1* gene has been demonstrated to be regulated by other epigenetic mechanisms such as microRNA expression [16,33].

Taking into account all these results, we can conclude that the methylation status of *CDH1* enhancers correlates with the gene expression levels and seems to have an important role in the gene regulation. Particularly in blood cells, where promoter methylation is absent, the presence of a specific pattern of methylation in the enhancers suggests that it could be a repression mechanism stronger than promoter methylation. This provides the rationale to investigate *CDH1* promoter/enhancer methylation in the gastric tissue of patients with suspected genetic predisposition to GC, since it could be an early event of the carcinogenesis process in HDGC *CDH1*-negative patients.

Moreover, *CDH1* hypermethylation could be a potential novel drug target for developing personalized therapies [34,35]. However, further studies on a larger number of patients with GC of different histotypes are necessary to confirm our results and to deeply understand the role of enhancer methylation in the regulation of *CDH1* gene.

## 4. Materials and Methods

### 4.1. Cell Lines

The study was performed on four commercial GC cell lines (SNU-1 ATCC Cat# CRL-5971, RRID:CVCL_0099, SNU-5 ATCC Cat# CRL-5973, RRID:CVCL_0078, KATO-III ATCC Cat# HTB-103, RRID:CVCL_0371, NCI-N87 ATCC Cat# CRL-5822, RRID:CVCL_1603) purchased from ATCC (American Type Culture Collection, Manassas, VA, USA), three GC cell lines (GK2, AKG, KKP) established and characterized at IRST laboratory, and one GC cell line (MKN-74) provided by the team of C. Oliveira, a co-author from the Institute of Molecular Pathology and Immunology of University of Porto (IPATIMUP, Portugal). The characteristics of the GC cell lines used in the present work are summarized in the Supplementary Table S4.

All the cell lines were maintained as a monolayer, incubated at 37 °C with 5% CO_2_ and subcultured weekly. For SNU-1, NCI-N87, and MKN-74, the culture medium was composed of RPMI-1640 Medium (ATCC) supplemented with 10% fetal bovine serum (FBS) (Euroclone, Milan, Italy). For SNU-5 and KATO-III, the culture medium was Iscove’s Modified Dulbecco’s Medium (ATCC) supplemented with 20% FBS. For AKG, GK2 and KKP, the culture medium was composed of DMEM/Ham’s F12 (1:1) supplemented with FBS (10%), glutamine (2 mM), non-essential amino acids (1%) and insulin (10 mg/mL) (Sigma-Aldrich, St. Louis, MO, USA). Cell lines were routinely tested for Mycoplasma using MycoAlert Mycoplasma Detection Kit (Lonza, Basel, Switzerland).

### 4.2. Blood Samples

The patients included in the study were selected by the Genetic Counseling service of the Area Vasta Romagna from 2010 to 2018 because they had a family and personal history of DGC and/or LBC.

In particular, the selection of patients was performed according to the first four criteria established by the International Gastric Cancer Linkage Consortium (IGCLC) in the 2015 HDGC guidelines, which must be valid among the first- and second-degree relatives: 2 GC cases regardless of age with at least 1 confirmed DGC (I); 1 case of DGC < 40 years (II); personal or family history of DGC and LBC with 1 diagnosed < 50 years (III); bilateral LBC or family history of 2 or more cases of LBC < 50 years (IV) [36].

All 20 patients underwent molecular testing for point mutations and copy number variations (CNVs) of the *CDH1* gene using Next-Generation Sequencing (NGS) and Multiplex Ligation-dependent Probe Amplification (MLPA), as reported in our previous work [37]. Of the 20 patients, 15 had developed DGC (range 22–39 years) and 5 had LBC (range 35–48 years). Patients’ characteristics are reported in Supplementary Table S5.

After the subscription of the informed consent, we collected the peripheral blood of these 20 patients and of 6 healthy controls.

### 4.3. Gastric Tissue Samples

Thirteen patients with sporadic GC submitted to surgical resection and classified by an expert pathologist, according to Lauren’s classification [3], were included in this study. Surgical samples were immediately cryopreserved after resection and fresh-frozen (FF) tumor tissue and matched normal gastric epithelium samples were stored as previously described [33]. The patients’ characteristics are summarized in Supplementary Table S6.

### 4.4. Nucleic Acid Extraction

The DNA was extracted from GC cell lines, peripheral blood samples and FF tumor and normal tissues using the QIAamp DNA Mini Kit (Qiagen, Hilden, Germany), according to manufacturer’s instructions. DNA was quantified by Qubit 4 Fluorometer (Thermo Fisher Scientific, Waltham, MA, USA).

Total RNA extraction was performed from GC cell lines and from FF tumor and normal tissues using TRIzol (Invitrogen, Carlsbad, CA, USA) in accordance with manufacturer’s instructions. Four micrograms of extracted RNA were treated with DNase and purified by using RNeasy MinElute Cleanup kit (Qiagen, Hilden, Germany), RNA was quantified by spectrophotometer Nanodrop-ND-1000 (Thermo Fisher Scientific).

### 4.5. Next-Generation Sequencing

The DNA libraries for the NGS analysis were prepared with Nextera Flex for Enrichment (Illumina, San Diego, CA, USA) and a custom probe panel (Integrated DNA Technologies, Coralville, IA, USA), and run on the Miseq sequencer (Illumina). Results were analyzed with Miseq Reporter software (Illumina) and further processed with a customized bioinformatics pipeline.

### 4.6. Identification of CDH1 Regulatory Regions

The promoter and the enhancers of the *CDH1* gene were identified through the UCSC Genome Browser [38] with the FANTOM5 tool [39,40] in the 2018 version. The enhancers were named from A to G starting from the most upstream one. The coordinates of *CDH1* promoter and enhancers are reported in the Supplementary Table S2.

### 4.7. DNA Methylation Analysis

DNA samples were treated with bisulfite using the Epitect Bisulfite kit (Qiagen, Hilden, Germany),

*CDH1* promoter and enhancers were amplified by standard PCR with the Multiplex PCR kit (Qiagen, Hilden, Germany), and the PCR products were sequenced by the BigDye Terminator v.3.1 cycle sequencing kit (Thermo Fisher Scientific) on an ABI-3130 Genetic analyzer (Applied Biosystems, Foster City, CA, USA), with the exception of enhancer C that was excluded from the analysis because of the absence of CpG sites. The primers used for amplification and sequencing were designed on the in silico bisulfite converted sequence and are reported in Supplementary Table S7.

### 4.8. Expression Analysis

The retrotranscription of RNA to cDNA was performed on 1000 ng of total RNA using the SuperScript II Reverse Transcriptase, random primers, dNTP mix and RNaseOUT (Invitrogen, Carlsbad, CA, USA) according to manufacturer’s instructions.

The *CDH1* expression analysis was performed by real-time PCR with TaqMan probes for *CDH1* gene as target (Hs01023894_m1, Thermo Fisher Scientific) and *B2M* gene as endogenous control for normalization (Hs00984230_m1, Thermo Fisher Scientific). The runs were performed on the 7500 Real-Time PCR System (Applied Biosystems).

Expression levels of the target genes were obtained by normalizing the results using the endogenous control *B2M*. For GC cell lines, the relative expression was quantified using the comparative 2^−∆Ct^ method. For the gastric tissues, the Fold Change (FC) values in gene expression were calculated using the 2^−∆∆Ct^ method [41,42]. An FC ≤ 0.50 and ≥1.5 were used as cut-off for downregulation and upregulation, respectively.

## 5. Conclusions

In conclusion, the results we obtained from the methylation analysis of *CDH1* promoter and enhancers demonstrate the existence of different methylation patterns within the regulatory regions of the gene and highlight that the methylation in *CDH1* enhancers could have an important role in the regulation of gene expression.

## Figures and Tables

**Table 1:** Results of *CDH1* expression on eight gastric normal (N) and tumor (T) tissues.

Gastric Tissue	2^−ΔCt^	Fold Change *CDH1*
06/07 N	2.412	0.056
06/07 T	0.135	
08/07 N	0.157	2.085
08/07 T	0.328	
09/07 N	0.210	0.279
09/07 T	0.059	
12/08 N	2.603	0.053
12/08 T	0.138	
05/09 N	1.338	0.028
05/09 T	0.037	
17/11 N	1.591	0.055
17/11 T	0.087	
18/11 N	2.297	0.042
18/11 T	0.097	
10/12 N	0.056	14.026
10/12 T	0.779	

**Table 2:** Summary of the results obtained on GC cell lines from *CDH1* sequencing, methylation and expression analyses.

Cell Line	*CDH1* Status ^1^	*CDH1* Promoter Methylation ^2^	*CDH1* Enhancer Methylation ^2^				*CDH1* Expression
			A	B	D	E/F/G	
NCI-N87	wt	−	−	−	+	+	normal
KATO-III	mut	−	−	−	−	+	normal
SNU-5	mut	−	+	+	+	+	low
SNU-1	wt	+	+	+	+	+	low
AKG	wt	−	−	−	+	+	normal
GK2	wt	+	−	+	+	+	low
KKP ^3^	mut	ND	ND	ND	+	+	low
MKN-74 ^4^	wt	−	−	−	+	+	normal

^1^ wt: *CDH1* wild-type; mut: *CDH1* pathogenic variant. ^2^ −: absence of methylation; +: presence of partial or full methylation. ^3^ The *CDH1* promoter and enhancers A/B are deleted in KKP cell line consequently the methylation analysis for these regions was not detectable (ND). ^4^ *CDH1* sequencing and expression analyses for MKN-74 cell line have been previously reported [30,31].

## Data Availability

The data presented in this study are available on request from the corresponding authors.

## Supplementary Materials

The following are available online at https://www.mdpi.com/article/10.3390/ph14050457/s1, Figure S1: Methylation analysis of *CDH1* promoter and enhancers from peripheral blood of 6 healthy individuals, Figure S2: Methylation analysis of *CDH1* promoter and enhancers from peripheral blood of 20 HDGC *CDH1*-negative patients, Figure S3: Methylation analysis of *CDH1* promoter and enhancers A and B on DNA samples from gastric normal (N) and tumor (T) tissues from 5 patients with sporadic GC, Table S1: Results of the *CDH1* variant analysis on 7 GC cell lines, Table S2: Genomic coordinates of *CDH1* regulatory elements identified by FANTOM5, Table S3: Results of *CDH1* expression on 7 different GC cell lines, Table S4: Characteristics of the 8 GC cell lines used in the study, Table S5: Clinical characteristics of the 20 HDGC *CDH1*-negative patients whose peripheral blood was analyzed, Table S6: Clinical characteristics of the 13 patients from whose gastric normal and tumor tissues were analyzed, Table S7: Sequences of the primers used for amplification and sequencing in the methylation analysis of *CDH1* regulatory regions on bisulfite-converted DNA.
