# Supplementary material for: Genetic and Epigenetic Alterations of CDH1 Regulatory Regions in Hereditary and Sporadic Gastric Cancer
Source: Pharmaceuticals (Basel). 2021 May 12;14(5):457. doi: 10.3390/ph14050457 (PMC8151134; doi:10.3390/ph14050457)
Supplement: Supplementary file 1 [file pharmaceuticals-14-00457-s001.zip › pharmaceuticals-1186938-supplementary.pdf]

pharmaceuticals

MDPI

Supplementary Materials

Supplementary Figure S1. Methylation analysis of *CDH1* promoter and enhancers from peripheral blood of 6 healthy individuals. ○ non-methylated CpG site; ◍ partially methylated CpG site; ● fully methylated CpG site.

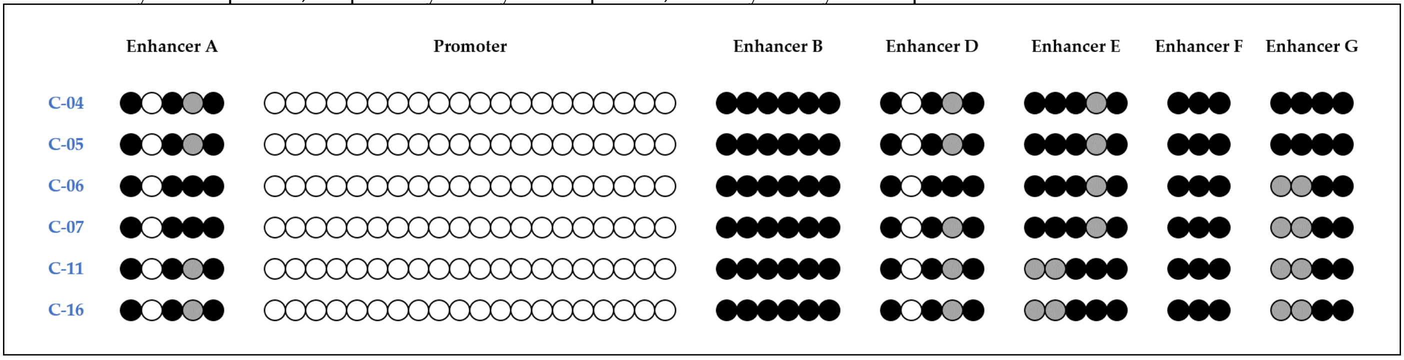

Supplementary Figure S2. Methylation analysis of *CDH1* promoter and enhancers from peripheral blood of 20 HDGC *CDH1*-negative patients (15 DGC patients in black and 5 LBC patients in violet). DGC: diffuse gastric cancer; LBC: lobular breast cancer; ○ non-methylated CpG site; ◍ partially methylated CpG site; ● fully methylated CpG site.

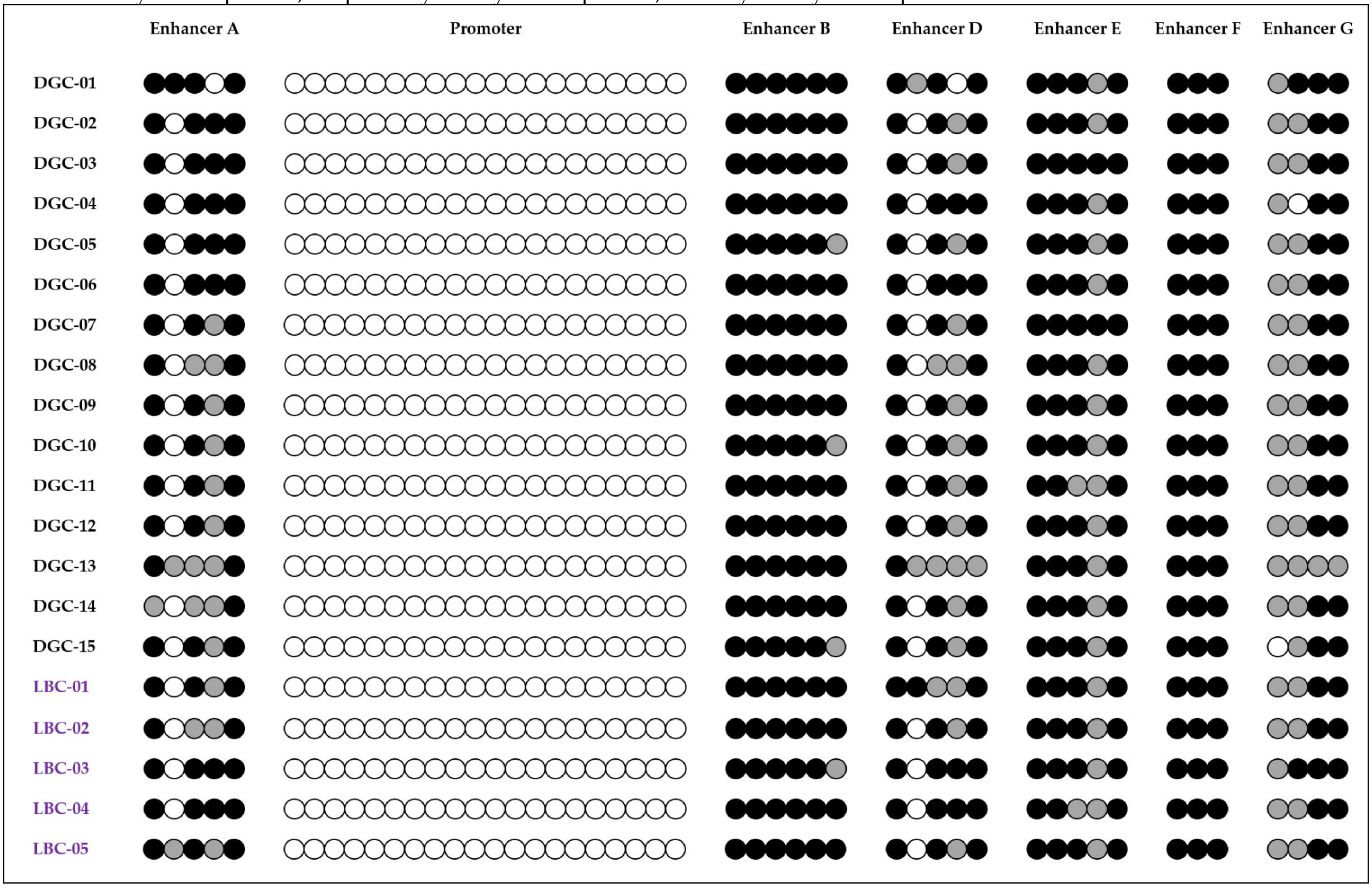

Supplementary Figure S3. Methylation analysis of *CDH1* promoter and enhancers A and B on DNA samples from gastric normal (N) and tumor (T) tissues from 5 patients with sporadic GC. IGC: intestinal gastric cancer; DGC: diffuse gastric cancer; Muc: mucinous gastric cancer; Mix: Mixed gastric cancer. ○ non-methylated CpG site; ◍ partially methylated CpG site; ● fully methylated CpG site; NA: not available.

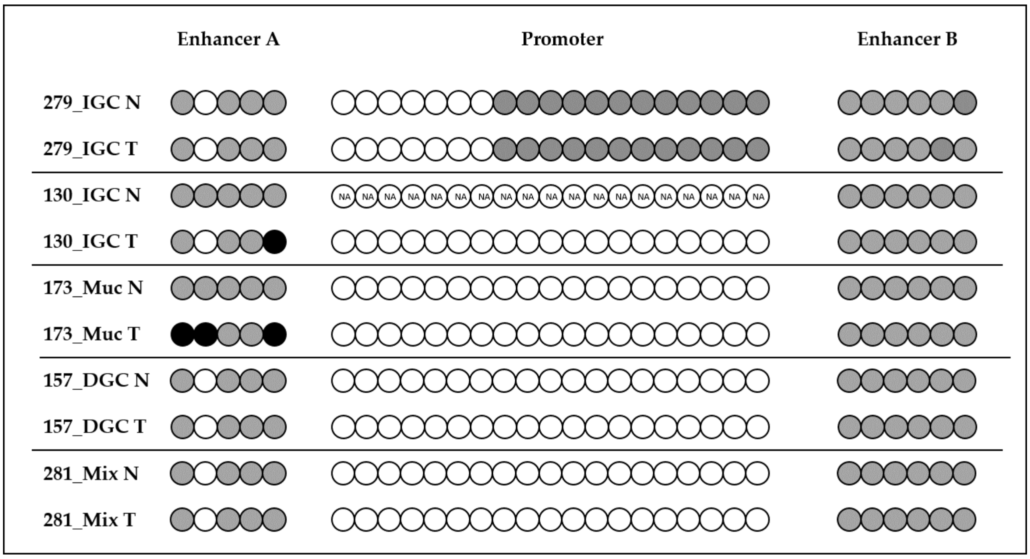

Supplementary Table S1. Results of the *CDH1* variant analysis on 7 GC cell lines.

GC cell line	*CDH1* status	cDNA	protein	VAF [1]
NCI-N87	wild-type	-	-	-
KATO-III	pathogenic variant	c.1008G>A	p.Glu336=	0.71
SNU-5	pathogenic variant	c.687+1G>C	p.?	1.00
SNU-1	wild-type	-	-	-
AKG	wild-type	-	-	-
GK2	wild-type	-	-	-
KKP	pathogenic variant	c.1-?_163+?del	p.?	1.00

[1] VAF: variant allele frequency.

Supplementary Table S2. Genomic coordinates of *CDH1* regulatory elements identified by FANTOM5.

CDH1 element	Genomic coordinates (hg38)	Size (bp)
Promoter	chr16: 68737132 - 68738441	1310
Enhancer A	chr16: 68732016 - 68732406	391
Enhancer B	chr16: 68744856 - 68745117	262
Enhancer C	chr16: 68745532 - 68745656	125
Enhancer D	chr16: 68760873 - 68761125	253
Enhancer E	chr16: 68770585 - 68770965	381
Enhancer F	chr16: 68784312 - 68784444	133
Enhancer G	chr16: 68791011 - 68791314	304

Supplementary Table S3. Results of *CDH1* expression on 7 different GC cell lines.

GC cell line	ΔC_t	$2^{-\Delta Ct}$
NCI-N87	1.810	0.285
KATO-III	3.081	0.118
SNU-5	10.879	0.001
SNU-1	13.622	$7.933*10^{-5}$
AKG	1.050	0.483
GK2	13.648	$7.789*10^{-5}$
KKP	13.446	$8.961*10^{-5}$

Supplementary Table S4. Characteristics of the 8 GC cell lines used in the study.

Cell line	Age	Sex	Ancestry	Origin	Tissue type	Reference
SNU-1	44	M	Asian	Primary gastric carcinoma	Poorly differentiated carcinoma	[1]
SNU-5	33	F	Asian	Ascitic effusion	Poorly differentiated carcinoma	[1]
KATO-III	55	M	Asian	Pleural effusion	Gastric carcinoma of the diffuse histotype[a] with signet-ring cells[b]	[2]
NCI-N87	-	M	-	Liver metastasis	Well-differentiated gastric carcinoma of the intestinal histotype[a]	[1]
GK2	60	M	European	Abdominal effusion	Poorly differentiated gastric adenocarcinoma of the intestinal histotype[a]	[3]
AKG	64	M	European	Pleural effusion	Infiltrating gastric cancer of the intestinal histotype[a]	[3]
KKP	63	M	European	Ascitic effusion	Poorly differentiated gastric carcinoma of the intestinal histotype[a]	[4]
MKN-74	37	M	Asian	Liver metastasis	Gastric tubular adenocarcinoma[b]	[5]

[a] Lauren classification [6]
[b] WHO classification [7]

Supplementary Table S5. Clinical characteristics of the 20 HDGC *CDH1*-negative patients whose peripheral blood was analyzed.

Patient ID	Sex	Diagnosis [1]	Age at diagnosis	Selection criteria [2]	*CDH1* status
DGC-01	F	DGC	36	I + II	WT
DGC-02	F	DGC	34	I + II	WT
DGC-03	F	DGC	32	II	WT
DGC-04	F	DGC	30	II	WT
DGC-05	M	DGC	33	I + II	WT
DGC-06	F	DGC	24	II	WT
DGC-07	M	DGC	32	II	WT
DGC-08	F	DGC	36	II	WT
DGC-09	F	DGC	36	II	WT
DGC-10	F	DGC	22	II	WT
DGC-11	M	DGC	39	II	WT
DGC-12	F	DGC	27	II	WT
DGC-13	M	DGC	33	I + II	WT
DGC-14	F	DGC	29	II	WT
DGC-15	F	DGC	39	II	WT
LBC-01	F	LBC	35	IV	WT
LBC-02	F	LBC	45	III	WT

LBC-03	F	LBC	47	III	WT
LBC-04	F	LBC	46	IV	WT
LBC-05	F	LBC	48	III + IV	WT

[1] Lauren classification [6]
[2] Selection criteria are according to the 2015 HDGC guidelines [8]

Supplementary Table S6. Clinical characteristics of the 13 patients from whose gastric normal and tumor tissues were analyzed.

Patient ID	Sex	Diagnosis [1,2]	Age at diagnosis
06/07	F	IGC	86
08/07	M	IGC	56
09/07	M	IGC	72
12/08	M	IGC	75
05/09	F	IGC	71
17/11	F	IGC	63
18/11	F	IGC	88
10/12	M	IGC	84
279_IGC	F	IGC	74
130_IGC	M	IGC	70
173_Muc	M	Mucinous	70
157_DGC	M	DGC	73
281_Mix	F	Mixed	84

[1] Lauren classification [6]
[2] WHO classification [7]

Supplementary Table S7. Sequences of the primers used for amplification and sequencing in the methylation analysis of *CDH1* regulatory regions on bisulfite-converted DNA.

***CDH1* element**	**Primer F (5'->3')**	**Primer R (5'->3')**	**Product size (bp)**
Promoter	GGTAGGTGAATTTTTAGTTAA	ACTCCAAAAACCCATAACTAACC	221
Enhancer A	GGGTGATGAATATTTGTGG	CACTTCCTAAATATTTTAACC	485
Enhancer B	GGAGGATTTGGTGTTGTTTG	CTAACAAATTCAAAATCATCCC	352
Enhancer D	GTTTTTTTAGAGTGGGGAG	CTACAATCCTACCAAACTC	330
Enhancer E	GGTAGTATATTTTAGAAGGATG	CTATCTCCTACACATCATTTC	504
Enhancer F	GGAGTTTTATATTTTTGTTGG	CCATACAAAATACAAAAAACC	272
Enhancer G	GTTGTTAAGAAGGGTATGG	CCAAAAACTTTATATAACAAAC	400

References

1. Park, J.G.; Frucht, H.; LaRocca, R. V; Bliss, D.P.; Kurita, Y.; Chen, T.R.; Henslee, J.G.; Trepel, J.B.; Jensen, R.T.; Johnson, B.E. Characteristics of cell lines established from human gastric carcinoma. *Cancer Res.* **1990**, *50*, 2773–80.
2. Sekiguchi, M.; Sakakibara, K.; Fujii, G. Establishment of cultured cell lines derived from a human gastric carcinoma. *Jpn. J. Exp. Med.* **1978**, *48*, 61–8.
3. Bertoni, L.; Zoli, W.; Mucciolo, E.; Ricotti, L.; Nergadze, S.; Amadori, D.; Giulotto, E. Different genome organization in two new cell lines established from human gastric carcinoma. *Cancer Genet. Cytogenet.* **1998**, *105*, 152–9.
4. Zoli, W.; Ricotti, L.; Lenzi, L.; Roncuzzi, L.; Zini, N.; Amadori, D.; Gruppioni, R.; Sensi, A.; Gasperi-Campani, A. Molecular genetics and in vitro sensitivity of a new human cell line, KKP, from a gastric adenocarcinoma. *Cancer Genet. Cytogenet.* **1998**, *105*, 43–9.

5. Hojo, H. Establishment of cultured cell lines of human stomach cancer origin and their morphological characteristics. *Niigata Igakkai Zasshi* **1977**, *91*, 737–763.
6. Lauren, P. The two histological main types of gastric carcinoma: diffuse and so-called intestinal-type carcinoma. An attempt at a histo-clinical classification. *Acta Pathol. Microbiol. Scand.* **1965**, *64*, 31–49.
7. International Agency for Research on Cancer *WHO classification of tumours of the digestive system*; Fred T. Bosman, Fátima Carneiro, Ralph H. Hruban, N.D.T., Ed.; 4th ed.; IARC, 2010; ISBN 9789283224327.
8. van der Post, R.S.; Vogelaar, I.P.; Carneiro, F.; Guilford, P.; Huntsman, D.; Hoogerbrugge, N.; Caldas, C.; Schreiber, K.E.C.; Hardwick, R.H.; Ausems, M.G.E.M.; et al. Hereditary diffuse gastric cancer: updated clinical guidelines with an emphasis on germline CDH1 mutation carriers. *J. Med. Genet.* **2015**, *52*, 361–74.
